# The Martyrdom Effect: When Pain and Effort Increase Prosocial Contributions

**DOI:** 10.1002/bdm.767

**Published:** 2011-12-22

**Authors:** Christopher Y Olivola, Eldar Shafir

**Affiliations:** 1 University of WarwickUK; 2 Princeton UniversityUSA

**Keywords:** martyrdom effect, self-sacrifice, charity, prosocial behavior, fundraising

## Abstract

Most theories of motivation and behavior (and lay intuitions alike) consider pain and effort to be deterrents. In contrast to this widely held view, we provide evidence that the prospect of enduring pain and exerting effort for a prosocial cause can promote contributions to the cause. Specifically, we show that willingness to contribute to a charitable or collective cause increases when the contribution process is expected to be painful and effortful rather than easy and enjoyable. Across five experiments, we document this “martyrdom effect,” show that the observed patterns defy standard economic and psychological accounts, and identify a mediator and moderator of the effect. Experiment 1 showed that people are willing to donate more to charity when they anticipate having to suffer to raise money. Experiment 2 extended these findings to a non-charity laboratory context that involved real money and actual pain. Experiment 3 demonstrated that the martyrdom effect is not the result of an attribute substitution strategy (whereby people use the amount of pain and effort involved in fundraising to determine donation worthiness). Experiment 4 showed that perceptions of meaningfulness partially mediate the martyrdom effect. Finally, Experiment 5 demonstrated that the nature of the prosocial cause moderates the martyrdom effect: the effect is strongest for causes associated with human suffering. We propose that anticipated pain and effort lead people to ascribe greater meaning to their contributions and to the experience of contributing, thereby motivating higher prosocial contributions. We conclude by considering some implications of this puzzling phenomenon. Copyright © 2011 John Wiley & Sons, Ltd.

“Endure and persist; this pain will turn to good by and by.” Publius Ovidius Naso (Ovid), Roman poet, (43 bc–17 ad)

A common view of motivation and behavior holds that humans (and other animals) are primarily driven to seek positive experiences (such as pleasure) and to avoid negative experiences (such as pain and effort). This hedono-centric “principle” was perhaps best articulated by Jeremy Bentham (1781/1988, p. 1), who famously stated: “Nature has placed mankind under the governance of two sovereign masters, pain and pleasure. It is for them alone to point out what we ought to do, as well as to determine what we shall do.”

Today, this assumption shapes lay views of human motivation (Heath, 1999; Kohn, 1999) and underlies many influential theories of behavior, including neoclassical economics and game theory (Bénabou & Tirole, 2003), learning and motivation theory (Hull, 1943), and optimal foraging theory (Stephens & Krebs, 1986).

Although this view may be appealing for its simplicity and consistency, it nonetheless paints a rather limited picture of human motivation and behavior. In doing so, it fails to account for a number of “hedonic puzzles”—situations where people deliberately avoid basic pleasures and actively seek out aversive experiences involving pain and effort. Examples include the near-uniquely human taste for painfully spicy foods (Rozin, 1999), the popularity of painful and effortful (not to mention dangerous) extracurricular activities such as mountaineering (Loewenstein, 1999), and other deliberate inflictions of pain (Berns, 2005; Walster, Aronson, & Brown, 1966). In fact, throughout history, cultures across the world have practiced rituals involving pain and/or effort (Glucklich, 2001; Smith, 2003).

One example of a striking modern-day hedonic puzzle is charitable fundraising; in particular, the challenging ways in which people choose to raise money. Some of the most popular and successful fundraisers involve considerable pain and effort (Symonds, 2005). Examples include charity walk-a-thons, marathons, and other fill-in-the-blank-a-thons for charity. That people exert so much effort to give up wealth stands in sharp contrast to the predictions of the standard labor economic model, which assumes that labor (i.e., effort) is a source of disutility and that people only work in order to *gain* resources, not give them away (Kaufman, 1999; Lane, 1992). Moreover, painful–effortful fundraisers are not limited to mere endurance events. Other variants include walking barefoot on burning coals (Firewalkers, 2004; Pub-goers, 2002) and broken glass (Barry, 2006; Birks, 2006), plunging into extremely cold water (Fenster, 2011; Kenderdine, 2011), and fasting for an extended period (Gardiner, 2007; Russell, 2004) to raise money for charity. Why are these painful–effortful fundraisers so popular?

Understanding the factors that motivate charitable giving has enormous implications for improving human (and nonhuman) welfare, yet much remains to be learned concerning these factors (Oppenheimer & Olivola, 2010). Studying charitable giving can also help us understand human altruism and collective behavior. This paper examines both charitable fundraising and prosocial contributions more broadly (i.e., beyond the charity context). In particular, we wish to shed light on a deep and theoretically interesting issue: the motivation people derive from pain and effort in the context of prosocial causes and how this can increase their voluntary contributions.

## PAIN AND EFFORT AS SOURCES OF VALUE

There is copious evidence that people derive meaning and value from their hard-earned accomplishments and the pains they endure on the road to goal achievement (Kaufman, 1999; Lane, 1992; Loewenstein, 1999). For example, people tend to value objects they have worked hard to earn over those obtained without effort (Lewis, 1965; Loewenstein & Issacharoff, 1994), contrary to the basic economic principle that the valuation of goods should be independent of the ways in which they were obtained (Loewenstein & Issacharoff, 1994; von Neumann & Morgenstern, 1947). Similarly, the more severe the process of initiation into a group, the more the group is subsequently liked (Aronson & Mills, 1959). This research suggests that overcoming pain and effort in order to achieve a goal adds meaning to the achievement and a kind of symbolic value to the associated outcome as a result. Meaning and symbolic value are important sources of utility, as evidenced by their impact on decision making (Ariely, Kamenica, & Prelec, 2008; Medin, Schwartz, Blok, & Birnbaum, 1999), yet most theories of choice have failed to incorporate them (Medin & Bazerman, 1999; Loewenstein, 1999).

The evidence thus seems to highlight one aspect of painful–effortful charity fundraisers that might render them especially popular and successful at drawing donations: if working hard (e.g., running a marathon) and suffering (e.g., walking barefoot on hot coals) to raise money for a charitable cause adds positive meaning to the fundraising process, then this may attract greater donations. In other words, people may contribute more to participate in painful-effortful fundraising events because the prospect of suffering to raise money for charity makes their donations seem more meaningful.

## MARTYRDOM AND PROSOCIAL CONTRIBUTIONS

Despite the evidence discussed above, many questions remain concerning the link between suffering for a cause and contributions to the cause.

First, the positive effect of pain–effort on valuation has mostly been documented when people are working to *obtain* resources and bring about outcomes that (they believe will) benefit them *personally*. To the best of our knowledge, no studies have examined how pain and effort affect the motivation to *give up* resources in order to benefit *others*. If anything, research shows that having to earn money and other resources (rather than obtaining them for free) *decreases* sharing and *reduces* prosocial contributions (Hoffman, McCabe, Shachat, & Smith, 1994; Kameda, Takezawa, Tindale, & Smith, 2002).

Furthermore, nearly all studies linking pain–effort to increased value have shown that people ascribe more value to things *after* they have experienced pain and exerted effort to obtain them. In contrast, very little research has examined whether the *prospect* of yet-to-be-experienced pain and effort can positively affect valuation. Although a number of theories anticipate that people will value objects and outcomes more once they have overcome an unpleasant experience to obtain them (Bem, 1967; Festinger, 1957), there is almost no theoretical work establishing a similar link between the *expectation* of *future* pain–effort and increased valuation. Yet such a link is necessary to explain why people might preemptively choose to donate more to charity events where they *anticipate* suffering for the target cause (i.e., *before* they experience any pain–effort).

Thus, a number of empirical and theoretical gaps need to be filled before we can understand the motivation to suffer for a prosocial cause and why the prospect of doing so often seems to increase prosocial contributions. In this paper, we attempt to fill these gaps by examining how the nature of the contribution process affects the likelihood and size of contributions. We hypothesize that people will perceive their contributions to be more meaningful when they are expected to overcome pain and effort in order to raise money for a prosocial cause. This leads to the central prediction of the paper, namely that making the contribution process painful and effortful will increase willingness to contribute prosocially, relative to an easy and enjoyable contribution process.

We call this phenomenon the “martyrdom effect,” as it essentially involves people suffering for a cause they believe in and care about. Martyrs and their acts of self-sacrifice are given special symbolic significance (Cormack, 2002; Fields, 2004). Analogously, people may ascribe additional meaning and value to the pain–effort that they anticipate enduring in order to raise money for a cause. Although the word “martyrdom” is commonly associated with religious fanaticism, the *Oxford English Dictionary* (2008) defines *martyr*, in its broadest sense, as “a person who undergoes death or great suffering for a faith, belief, or cause,” and *martyrdom* as “the act of becoming or the condition of being a martyr.” Combining these definitions, *martyrdom* is simply the act of suffering for a cause.

This definition is particularly suitable because the focus on a cause (i.e., the reason one is suffering) is critical in our account. We do not expect that suffering will increase contributions on its own. Instead, we predict that the anticipation of pain and effort will only be meaningful if they are perceived to be necessary for promoting a valued cause (Thompson & Bunderson, 2003). Once a person is made aware of an easy alternative (a painless–effortless way to contribute), martyrdom will lose its appeal and seem pointless, even foolish. We examine this hypothesis in Experiment 1. Moreover, in the account we propose, pain and effort increase willingness to contribute by making the experience and act of contributing seem more meaningful, as we show in Experiment 4.

We conducted five experiments to test the hypothesis that the prospect of pain and effort can increase contributions to a prosocial cause. Specifically, we compare people's willingness to contribute when the contribution process is expected to be painful–effortful versus easy–enjoyable.

## EXPERIMENT 1A

Experiments 1A and 1B compared people's willingness to contribute to a charity fundraiser when the donation process is easy and enjoyable versus painful and effortful. We chose an outdoor charity picnic as the easy–enjoyable event and a 5-mile charity run as the painful–effortful event. Both events are organized efforts to raise money and involve being outdoors, surrounded by many other donors who are part of the same fundraising process. These fundraisers therefore share many features, including the sense of teamwork and the pleasure of being outdoors. However, a picnic is a pleasant and easy-going experience for most people, whereas running long distances is typically painful and effortful. Of course, many people regularly run for a variety of reasons (e.g., as a form of exercise), so running is by no means a strange or uncommon behavior for someone to willingly engage in. Still, given that runners typically have access to exercise machines, parks, tracks, and running mates, it is unclear what additional benefits they would derive by running for charity when they could make a donation online, send a check, or participate in a charitable event that costs less to organize and requires no effort. From this perspective, there is no reason to predict that even the most athletic person would be motivated to donate *more* money when the process is painful and effortful.

According to the martyrdom hypothesis, however, participants will be willing to contribute more to a cause when the fundraising process involves running 5 miles (a painful–effortful experience) than when it involves attending a picnic (an easy–enjoyable experience). By itself, this tendency need not be inconsistent with neoclassical economic theory, which allows decision makers to have a wide variety of tastes (Becker, 1993; Lewin, 1996). A willingness to donate more to participate in a charity run (vs. a charity picnic) could reveal a general preference or “taste” for challenging fundraisers. According to this “taste for painful benevolence” account, people should prefer the charity run even when they are explicitly provided with an easier alternative. If, instead, what motivates people to donate more when considering the charity run in isolation is the meaningfulness or added value of a hard-earned contribution to an important cause, then we might make a different prediction: the pain and effort invested in the donation process would become meaningless when an alternative option to contribute painlessly is made available. The explicit addition of an easy–enjoyable alternative makes suffering for a cause seem like a pointless act that provides no additional value.

To illustrate this point, consider the following scenario.^1^ Imagine that a close friend is sick and too physically weak to perform her chores. As a result, her sink is piled to the ceiling with dirty dishes that she has been unable to clean. During a visit, you decide to surprise her by washing all of her dishes—a long and difficult process that takes you an hour of hard scrubbing, washing, rinsing, and drying to complete. Just as you finish putting away the last clean dish, your friend enters the kitchen to discover your surprise, which she can clearly see involved a good deal of time and effort on your part. Consider how happy and proud this would make you feel. Now imagine that your friend then reveals that (unbeknownst to you) her kitchen is equipped with a brand new dishwasher, which (had you known of its existence) you could have used instead of washing her dishes by hand, thereby saving yourself a lot of time and effort while ultimately yielding the same clean dishes. Imagine how you would feel upon learning that all your efforts could have been easily avoided.

According to the martyrdom hypothesis, when people have to endure pain and exert effort for a cause (e.g., helping a sick friend or a charity), their contributions seem more meaningful, *unless* they are made explicitly aware of a painless alternative, which then trivializes their (potential) efforts. For rational choice theory, in contrast, a willingness to donate more when the fundraising process is painful–effortful (compared with easy–enjoyable) implies a preference for combining pain–effort and donations. Just as being willing to pay more for A than B (when these options are presented separately) implies a preference for A, a willingness to pay more to participate in a painful–effortful event than an easy–enjoyable one implies a preference for the former type of fundraiser. Accordingly, people should consistently prefer participating in the painful–effortful fundraiser over the easy–enjoyable one, even when the two are explicitly compared. To test these opposing hypotheses, we asked a separate group of participants to consider the two fundraisers—the charity picnic and the charity run—simultaneously. Contrary to a “taste for painful benevolence” explanation but in line with the martyrdom hypothesis, we predicted that participants would predominantly prefer the picnic to the 5-mile run when the two are presented jointly but would donate more to run 5 miles when these options are presented in isolation.

### Method

#### Participants

A total of 136 US undergraduate students (43% female, 47% male, and 10% not reporting gender) participated for course credit or compensation.

#### Procedure

This experiment was conducted in the months following the 2004 tsunami in Southeast Asia, which killed more than 225,000 people in 11 countries and caused huge amounts of damage and injury. A short questionnaire asked participants to imagine that a nonprofit organization was sponsoring a charity fundraiser to raise money for the tsunami victims.

Using two different hypothetical scenarios (see Appendix) in a between-subjects design (the “separate evaluation” conditions—see Hsee, Loewenstein, Blount, & Bazerman, 1999), we manipulated the donation process. In both versions of the scenario, attending the event was contingent on making a donation (any amount greater than $0), and 5000 people were expected to attend. Participants were assigned to one of the two conditions in alternating order (because of chance or experimenter error, five more participants were assigned to one condition than the other). One group was assigned to the easy–enjoyable fundraiser scenario, in which the cost of donating (in terms of effort and pain) was low, whereas a second group was assigned to the painful–effortful fundraiser scenario, which involved a high (physical) cost of contributing. Participants assigned to the easy–enjoyable fundraising condition were told that the fundraiser was a charity picnic and that all donations made by picnic attendees would be matched by the organization. Participants assigned to the painful–effortful fundraising condition were told that the fundraiser was a 5-mile charity run and that, in order to enter the run, they would have to make a donation that would only be matched if they successfully completed the run.

Following the scenario, the questionnaire asked participants two simple questions designed to measure their willingness to donate: (i) Would they attend the fundraiser? (ii) If so, how much would they donate to attend? Answering “no” to the first question was coded as a donation of $0. Within each condition, donations that fell outside an interval equal to three times the inter-quartile range (3 × IQR) were labeled as outliers and excluded from the analysis. This led to the exclusion of data from seven participants who reported exceptionally large donations.^2^ Thus, our final sample consisted of 96 participants.

A third group of 33 participants was administered the “joint evaluation” version of the questionnaire. In this scenario, the organization was said to be simultaneously sponsoring three different kinds of fundraisers to aid tsunami victims: the picnic and 5-mile run described above as well as a 20-mile charity walk. As with the separate-evaluation conditions, the scenario explained that attending an event was contingent on making a donation, that donations would be matched upon completing the event, and that 5000 people were expected to attend in all three cases. The order in which fundraisers were listed was counterbalanced across participants. The questionnaire instructed participants to imagine that they “were going to attend one of these events,” and they were asked to “rank the events in order from most appealing (most likely to attend) to least appealing (least likely to attend).”

### Results and discussion

**Figure 1:**
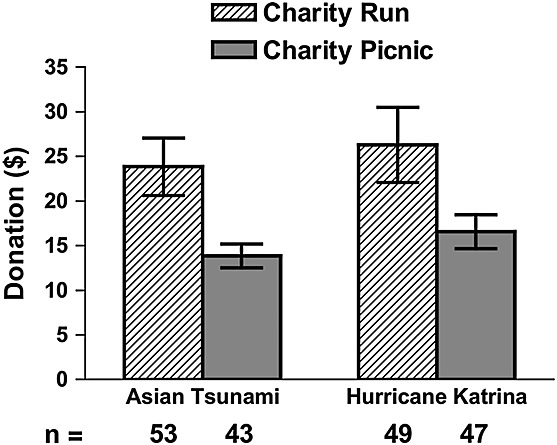
Mean donations as a function of fundraising condition in Experiment 1A (bars on the left) and Experiment 1B (bars on the right). The means include nonparticipation responses, coded as $0 donations. Numbers underneath the bars indicate the sample size in each condition. Error bars represent ±1 standard error

For the separate-evaluation scenarios, the reported likelihood of participating in the fundraiser was comparable across conditions: 86% accepted to participate in the picnic, and 76% in the 5-mile run (*χ*^2^(1) = 1.67, ns). Yet, our main hypothesis was supported: the average amount donated^3^ (including those refusing to participate—i.e., $0 donations) differed between fundraiser types. The mean amounts donated in each condition are presented in Figure 1. Given the highly skewed distribution of donations, a parametric test might not be appropriate, so we used a random permutation test with 5000 iterations, in which the permutation statistic was the difference in average donations between conditions. The permutation test revealed that participants in the painful–effortful fundraising condition offered to donate more (*M* = $23.87) than participants in the easy–enjoyable fundraising condition (*M* = $13.88), *p*_perm_ = .004. A *t*-test for unequal variances provided the same conclusion, *t*(68.57) = 2.87, *p* < .006, *d* = .70. Adding pain and effort (a 5-mile run) to the charitable contribution process increased participants' willingness to donate.

In contrast to the preferences implied by responses in the separate-evaluation conditions, a significant majority (76%) of participants in the joint-evaluation condition ranked the charity picnic above the charity run in terms of appeal (i.e., likelihood of attending), *χ*^2^(1) = 8.76, *p* < .004, *ϕ* = .52. In support of our hypothesis, when both the easy and the painful events were simultaneously available, participants predominantly chose the painless–effortless fundraising option. This is contrary to an exotic taste for painful fundraising.

In an effort to replicate our results, we ran a second study (Experiment 1B) that was nearly identical to the first except for two differences: in the joint-evaluation condition, participants considered only two options (charity run versus charity picnic) and chose one rather than rank-ordering them. The scenario also involved a different charitable cause: the fundraiser was described as collecting donations for victims of Hurricane Katrina.

## EXPERIMENT 1B

### Method

#### Participants

A total of 140 US undergraduate students (66% female, 28% male, and 6% not reporting gender) participated for compensation or course credit.

#### Procedure

The data were collected in the months following the 2005 devastation caused by Hurricane Katrina on the Gulf Coast. The procedure was identical to the one used in Experiment 1A.

Fifty participants were assigned to the easy–enjoyable fundraiser (charity picnic) condition, and another 50 to the painful–effortful fundraising (charity run) condition.

Removal of outliers using the 3 × IQR rule led us to exclude data from four participants. Our final sample consisted of 96 participants.

A third group of 40 participants considered a joint-evaluation scenario, in which a charity picnic and charity 5-mile run were simultaneously being organized to aid Katrina victims. We counterbalanced the order in which these fundraisers were listed. Participants were asked to imagine that they could only attend one of these two events and to select the one they would prefer attending. They could also indicate that they preferred not to attend either event. Only one participant chose this option, and his data were excluded from the analysis.

### Results and discussion

As with Experiment 1A, the reported likelihood of participating in a fundraiser was similar across separate-evaluation conditions: 89% accepted to participate in the picnic and 78% in the 5-mile run (*χ*^2^(1) = 2.41, ns). However, the average amount donated (including nonparticipants contributing $0) differed between conditions. As Figure 1 shows, we replicated the results of Experiment 1A. Participants in the painful–effortful fundraising condition offered to donate significantly more (*M* = $26.33) than those in the easy–enjoyable fundraising condition (*M* = $16.59), *p*_perm_ < .03, *t*-test for unequal variances: *t*(66.85) = 2.10, *p* < .04, *d* = .51.

In contrast, a significant majority (68%) of participants in the joint-evaluation condition preferred attending the charity picnic to the charity run, *χ*^2^(1) = 4.90, *p* < .03, *ϕ =* .35.

These results corroborate those of Experiment 1A. The shift in preferences from separate to joint evaluation suggests that the presence of an easy alternative for donating (the picnic) stripped away the meaningfulness of the painful–effortful contribution process (the 5-mile run), leading participants to opt for plastic tableware over muscle cramps. These findings favor the martyrdom hypothesis over a “taste for painful benevolence” account, because participants inconsistently indicated that they valued the painful–effortful fundraiser more in separate evaluation (given the amounts they were willing to pay to participate) but less in joint evaluation (given their choices).

## EXPERIMENT 2

Despite yielding results with fairly large effect sizes, Experiment 1 suffered from two potential flaws: first, the donation decisions were hypothetical, neither involving real pain nor actual contributions of money. An important question, then, is whether these results would replicate with real money and pain. Second, the charity run in the painful–effortful condition was roughly modeled after those organized in real life. Participants' donation decisions may have been affected by features that are unique to these fundraisers, such as the growing popularity of charity endurance events, the culture of endurance sports, the public nature of the fundraising effort, and the fact that running is a relatively common behavior. We were also interested in whether the martyrdom effect extends to non-charity contexts.

To resolve these issues, participants in Experiment 2 made decisions about how much to contribute (financially) to a prosocial cause in a novel context involving real pain and money. Specifically, they played a *public goods game* (Camerer, 2003; Ledyard, 1995), in which they each anonymously divided a sum of money between themselves (a selfish cause) and the group (a prosocial cause). This game is designed to create a conflict of interest between the group, which, as a whole, benefits from prosocial allocations, and the individual players, who each stand to gain more by keeping their money, regardless of what other players do. For half the participants, contributing to the group was contingent on performing a painful task: keeping both hands immersed in extremely cold water for 60 seconds. In accordance with the martyrdom effect, we predicted that participants who would have to suffer through this cold pressor task in order to contribute would allocate more of their budget to the group than would those for whom contributing would be pain-free. We also examined their beliefs about what other players would do, both within their own game and more generally among all students who had played the same game.

### Method

#### Participants

Thirty-six US undergraduate students (64% female) participated for course credit.

#### Procedure

Participants were each given a budget of $5 to divide between themselves (personal earnings) and the public pool (shared winnings). Participants could allocate any amount between $0 and $5 (in increments of $0.25) to the public pool, with the remainder allocated to themselves. All the money allocated to the public pool was doubled and then redistributed evenly to all the players in the game, regardless of how much or how little each had allocated to the public pool. Money kept (i.e., not contributed) by a participant did not double in value and was simply part of his/her final payoff.

Participants were run in groups of three to five. They were brought into a large room where they were simply told that they would be playing a “strategic interactive decision-making game.” At this point, they were instructed not to communicate with each other in any way for the duration of the game. An experimenter then led one participant (at a time) to another, smaller room, in a separate part of the building. A second experimenter stayed in the larger room to make sure that the remaining participants did not communicate. While waiting in the larger room, participants completed unrelated surveys. In the smaller room, the first experimenter read a script to the participant, which explained the rules of the game for the condition they were assigned to (see below), including any costs of contributing money to the public pool. These instructions were designed to ensure that participants clearly understood these rules before they made their allocation decisions. No deception was used in this experiment, and participants were fully informed about every aspect of the game that they were assigned to *before* they made any decisions. Only three pieces of information were hidden from participants: (i) the allocation decisions and final payoffs of the other players, (ii) the purpose of the experiment, and (iii) the existence of another treatment condition. After the experimenter had finished reading the instructions, the participant was allowed to ask any clarification questions about the rules of the game. Once the participant indicated that he/she understood the rules, the experimenter gave the person a sheet on which to indicate how much of his/her $5 budget he/she wanted to allocate to the public pool and how much he/she wanted to keep for him/herself. Participants were given as much time as they wanted to make their allocation decisions. Bringing participants one at a time into a separate room to make their allocation decisions ensured anonymity.

Participants in each session were arbitrarily assigned, as a group, to one of two experimental conditions (or game types). Participants in the control condition (*n* = 18) played the standard version of the public goods game, as described so far. The rules and instructions for the painful contribution condition (*n* = 18) were similar to those used in the control condition with one key difference: participants had to endure an aversive experience if they chose to allocate *any positive* amount of money to the public pool. We used a well-established method for inducing pain: the cold pressor task (von Baeyer, Piira, Chambers, Trapanotto, & Zeltzer, 2005), which involves immersing a part of the body in painfully cold water for an extended period of time. Participants in the cold pressor condition were informed that, in order to allocate money to the public pool, they would have to simultaneously keep both their hands submerged (up the wrists) in 10°C (50°F) water for 60 seconds.^4^ Failure to do so meant that one's contribution to the public pool would not double in value. Participants in the painful contribution condition were thus free to avoid the cold pressor task by simply choosing to keep all the money for themselves. These participants thus had an additional (and visceral) incentive to keep all their money. Participants in the cold pressor condition made their allocation decisions *before* experiencing the cold pressor task, although they were given the option of quickly sampling the water with their hand before deciding. Those who chose to allocate any part of their budget to the public pool then kept their hands in the water while the experimenter timed them. After the 60 seconds expired, they were given paper towels to dry their hands so that no evidence of the cold pressor task remained for the other players to see. The experiment was also designed so that participants who chose to keep all the money for themselves would stay in the room for an extra minute (timed by the experimenter), thereby equating the time spent in the smaller room by participants who did and did not endure the cold pressor task (every participant in this condition chose to allocate some of their budget to the public pool).

After a participant completed this part of the experiment, the experimenter reminded him/her not to communicate his/her decision to the other players. The participant was then brought back to the larger room, where he/she completed a questionnaire about the game, while the next player in the group was led to the smaller room. This questionnaire asked participants a series of questions about the way they made their allocation decisions and what they thought other players would do. In particular, participants were asked to estimate, both for their group and for all students at their university who had played the same game recently, the proportion of players who had contributed some money to the public pool and the average amount allocated to the public pool by those who had contributed.

Each participant's final payoff was equal to the money kept (i.e., not contributed) plus his/her equal share of the money in the public pool (which had doubled in value). Payoffs were thus a function of both one's allocation decision and the allocation decisions of all other players (which, combined, determined the size of the public pool). Each participant was given a sealed envelope containing his/her final payment (in cash) and a sheet that explained the purpose of the experiment.

### Results and discussion

**Figure 2:**
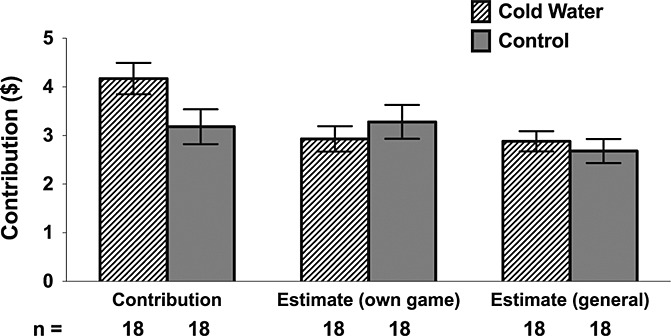
Mean contributions (among all players) and mean estimated contributions (among those contributing) for each condition in Experiment 2. Numbers underneath the bars indicate the sample size in each condition. Error bars represent ±1 standard error

The number of participants in a session (*n* = 3, 4, or 5) had no significant effect on mean contributions, both according to a one-way analysis of variance (ANOVA) (*F*(2, 33) < .3) and a nonparametric Kruskal–Wallis test (*χ*^2^(2) < .4). Because returns on contributions to the public pool decrease with more players, contributions should decrease as group size increases. Yet we found no correlation between these two variables (*r*(36) = .06, ns). We therefore collapse across group size for all remaining analyses. All but one participant (in the control group) allocated some of their budgets to the public pool. Figure 2 presents, for each condition, the mean amounts that participants contributed and their mean predictions of others' contributions. Participants in the cold pressor condition allocated nearly a dollar more to the public pool than participants in the control condition (*M* = $4.17 vs. $3.18, which represented 83% vs. 64% of their total budget). This difference was significant, *p*_perm_ < .03; *t*(34) = 2.04, *p* < .05, *d* = .70. A difference of $1 is quite large when compared with the largest possible difference between conditions, which was only $5. In fact, participants in the cold pressor condition were more likely than control participants to contribute their *entire* budget to the public pool (67% vs. 28%, *χ*^2^(1) = 5.46, *p* < .02, *ϕ =* .39). As a result of their larger contributions, players in the cold pressor condition obtained higher final payoffs than those in the control condition (*M* = $9.38 vs. $8.18, *t*(34) = 2.07, *p* < .05, *d* = .71).

In contrast, there were no significant differences, between the cold pressor and control conditions, in participants' beliefs concerning the proportion of players who would allocate to the public pool, either in their group specifically (*M* = 81% vs. 71%, *t*(34) = 1.50, ns) or generally (*M* = 81% vs. 77%, *t*(34) < 1). Participants in the cold pressor and control groups also reported similar beliefs regarding the mean allocations of players who contributed to the public pool, both in their own group (*M* = $2.93 vs. $3.28, *t*(34) < 1) and generally (*M* = $2.88 vs. $2.68, *t*(34) < 1). Furthermore, within-subject one-sample *t*-tests, comparing amounts contributed with beliefs about other contributors' allocations, revealed that the average participant in the cold pressor condition believed he/she had allocated more to the public pool than other contributors, both in his/her group (*M_Δ_* = $1.24, *t*(17) = 4.55, *p* < .001) and in general (*M_Δ_* = $1.29, *t*(17) = 5.48, *p* < .001). For control participants, however, no significant differences were found, either regarding their own group (*M_Δ_* = −$0.10, *t*(17) < .3) or all other players in the same condition (*M_Δ_* = $0.50, *t*(17) = 1.75, ns).

In support of our hypothesis, we found that participants contributed more when doing so was painful (and mentally effortful) than when it was neutral (i.e., devoid of pain). This is a strong demonstration of the martyrdom effect because real pain and money were at stake and contributions were made anonymously (i.e., participants were not aware of each others' decisions), removing incentives to respond untruthfully. By creating a context that abstracted away from real-world charity events, we were able to eliminate potential confounds typically associated with these events, such as their popularity, their cultural aspects, the public nature of fundraising efforts, and the fact that running, walking, swimming, and biking are relatively common behaviors. At the same time, this experiment maintained the key feature of interest: contributions to a prosocial cause (the group) that are contingent on pain–effort.

This experiment also allowed us to consider an alternative explanation for the martyrdom effect: that decision makers assume that a cause must be worth contributing to if others are willing to suffer for it and that the existence of a painful fundraiser implies that others must, in fact, be willing to do so (see Gneezy & Rustichini, 2000). An inferential account of this type entails that participants in the cold pressor condition, who tend to allocate more, would expect other players in their condition to do the same. Instead, we found that beliefs about others' allocations were similar in both conditions, which is inconsistent with the above hypothesis.

## EXPERIMENT 3

One possible explanation for our results is that participants are simply using the amount of pain or effort to be experienced in the contribution process as a determinant of how much they should contribute, substituting this cue for some other, more relevant but less accessible measure of “contribution worthiness.” This relatively simple “attribute substitution” strategy has been proposed to explain the process underlying many of the heuristics studied in the judgment and decision-making literature (Kahneman & Frederick, 2002). Often, when a relevant cue is inaccessible or difficult to evaluate, people will rely on another cue that is less relevant but easier to access, easier to evaluate, and seemingly related to the judgment of interest. In the case of prosocial contributions, potential contributors might rely on the amount of pain or effort involved as a proxy for the importance of the cause. If no other relevant cues are accessible (and potential contributors have difficulty evaluating how much they care about the cause), then they might use the level of pain–effort involved as a substitute for how much they should value contributing. In the case of a charity run, for example, the distance run may provide an indirect measure of the amount of pain–effort involved and, as a result, might influence their willingness to donate.

We followed Kahneman and Frederick's (2002) proposed method for testing the attribute substitution hypothesis: using hypothetical scenarios similar to those in Experiment 1, we varied the distance to be run to see how this would affect preferences for donating. If contribution rates are driven by a simple attribute substitution process (with amount of pain–effort as the replacement cue), then we would expect to see a positive correlation between the distance of the run and the amount donated. Finally, to ascertain whether people are even able to differentiate between running-distances as correlates of pain and effort, we asked other participants to report their asking price for having to run those various distances. If people are sensitive to distance when evaluating the pain of running, then we would expect their asking price (the subjective cost of running) to be correlated with the distance of the run.

### Method

#### Participants

A total of 202 US undergraduate students (57% female) participated for compensation or course credit.

#### Procedure

Participants completed one of two versions of a questionnaire involving hypothetical scenarios and choices. One version (the donate condition) was nearly identical to the painful–effortful (charity run) fundraising condition in Experiment 1, except that the goal of the run was to raise money for research to cure Alzheimer's disease. In addition, the distance of the charity run was varied between participants and ranged from 1 mile up to 20 miles, in 1-mile increments. For this first condition, we assigned five participants to each distance. The dependent variable was the amount of money that respondents were willing to donate to run, with a decision to not donate coded as $0. In the second version (the cost condition), participants reported the smallest amount of money they would have to be paid to run a certain number of miles that again varied between 1 and 20 miles (in 1-mile increments) in a between-participants design. Again, we assigned five participants to each distance. The dependent variable in this condition was the amount of payment that participants required in order to run the distance assigned to them in the survey. Overall, we assigned 100 and 102 participants to the donate and cost conditions, respectively (experimenter error led to two extra participants being assigned to the cost condition).

### Results and discussion

Following the approach proposed by Kahneman and Frederick (2002), we measured, for each condition, the correlation between running-distance and the rank of either the amount donated or the subjective cost for that distance. We report both the correlation based on individual responses and the correlation based on the mean response for each distance. We found no significant correlations between the distance of the charity run and the rank of the amount donated: *r*(100) = −.10, ns and *r*(20) = −.29, ns, for the individual-based and mean-based correlation, respectively. Even after controlling for age and gender, we found that distance did not significantly predict donation rank: standardized coefficient: *β* = −.09, *t*(96) < .9. One possible reason for the lack of correlation between distance and donation rank might be that this relationship is curvilinear rather than linear. To test for this possibility, we regressed donation rank on age, gender, a linear distance term, and a quadratic distance term (obtained by centering distance, then squaring it). The quadratic distance term did not significantly predict donation ranks: *β* = −.12, *t*(95) = 1.18, ns. Thus, participants do not seem to be using running-distance (i.e., a simple measure of pain–effort) as a cue to determine how much they should donate. The question that remains, then, is whether participants are not using distance because they are insensitive to this cue when they evaluate it separately (because distance was manipulated between participants) or because they do not choose to use this cue even if they are sensitive to it in separate evaluation. To determine whether participants were sensitive to distance as a cue and correlate of pain–effort, we examined the relationship between running-distance and the reported cost of running. Here we found a significant correlation between distance and cost rank: *r*(102) = .59, *p* < .001 and *r*(20) = .83, *p* < .001 for the individual-based and mean-based correlation, respectively. Distance predicted cost rank, even after controlling for age and gender: *β* = .59, *t*(98) = 7.21, *p* < .001. Clearly, participants are able to use distance as a substitute for pain–effort (presumably, the main variable of interest when evaluating the cost of running), even in separate evaluation.

These results show that, although participants are sensitive to running-distance (even when evaluated in isolation), they do not use this cue when determining how much to donate. The effect of pain and effort on contributions to prosocial causes does not, therefore, seem to be the result of a simple attribute substitution process that relies on the amount of pain–effort involved (or the distance to run). Rather, if participants are relying on a cue to evaluate contribution worthiness, it seems to be the presence or absence of pain–effort, not so much its specific quantity.

## EXPERIMENT 4

In Experiment 4, we examined whether feelings of meaningfulness associated with completing a painful–effortful fundraiser might drive the martyrdom effect. As with Experiment 1, participants reported their willingness to participate in and contribute to a charity fundraiser that was either painful–effortful (a 5-mile run) or easy–enjoyable (a picnic). In addition, they reported how meaningful the experience of participating and the act of giving would be to them. We predicted that the perceived meaningfulness of completing a painful–effortful fundraiser versus an easy–enjoyable one would mediate the relationship between the nature of the fundraiser and the amount that participants would be willing to give to the charitable cause.

### Method

#### Participants

Five hundred sixty-four British residents were recruited through the Maximiles online survey service (http://www.maximiles.co.uk; see Reimers, 2009, for additional details) and participated in exchange for compensation. Only those fluent in English, aged 18–50 years, were recruited. Prior to analyses, we discarded the responses of 69 participants who either failed one of our “catch” questions (details below), spent less than a minute completing the entire survey, and/or had an IP address that was identical to one belonging to a previous participant (and therefore might indicate repeat survey taking). We also discarded data from one additional respondent who reported that he would both participate in the fundraiser and donate £0 to do so (an inconsistent pair of responses given the instructions). These filters were used to help ensure that participants took the study seriously. Finally, we discarded responses from 135 participants who reported having a medical condition or other physical constraint that would prevent them from participating in an outdoor picnic and/or a 5-mile run (this filtering was applied regardless of condition assignment). After these eliminations, our sample consisted of 359 participants (48% female) who were 19–50 years old (*M* = 38.37, *SD* = 6.70). Of these respondents, 95% reported having previously donated money to charity, 31% reported that they liked to run or jog as a form of exercise, and 24% reported having previously participated in a charity run or charity marathon.

#### Procedure

The experiment was conducted online, via a web questionnaire. Participants were sent an e-mail containing a link to the study and an invitation to participate in exchange for compensation. The first page of the web questionnaire briefly introduced the study and asked participants to complete the survey attentively and on their own. Participants were then presented with a scenario about participating in a fundraiser for charity before being asked a series of questions about the scenario. The scenarios in this study were identical to those in Experiment 1 (in the separate-evaluation conditions), except that the fundraiser was described as raising money for victims of war and genocide, and donation amounts were in British pounds (£) rather than US dollars. Furthermore, in addition to reporting their willingness to participate and donate, participants rated the meaningfulness of participating in the fundraiser. Specifically, they were presented with the following instructions and questions:

Please take a moment to imagine [attending the picnic/completing the run] and knowing that, as a result, your donations have been matched and will go toward aiding victims of war and genocide.
How meaningful (to you) would this experience be?
How meaningful (to you) would your participation in the event be?
How meaningful (to you) would your contribution be?

Participants responded to each question using a 1–10 scale (with 1 = “Not at all meaningful”; 10 = “Very meaningful”).

The experiment consisted of a 2 × 2 between-subjects design, with participants randomly assigned to one of the four conditions. As with Experiment 1, we varied whether the fundraiser was an outdoor charity picnic or a 5-mile charity run. We also varied whether participants were first asked their willingness to participate and donate (followed by the meaningfulness ratings) or first asked the meaningfulness rating questions (followed by the participation and contribution questions).

Following the scenario, participants reported their age, gender, whether they had ever donated money to charity before, whether they liked to run and/or jog as a form of exercise, and whether they had ever participated in a charity run or charity marathon before. They were also asked to indicate whether they had any medical conditions or other physical constraints that would prevent them from participating in an outdoor picnic or a 5-mile run (*all* participants, regardless of condition assignment, were asked *both* about their ability to picnic *and* to run). Finally, participants were asked a series of “catch” questions designed to identify respondents who were not engaged in the study. One question asked them to report the year they were born. We then calculated their implied age and compared it with the age they had previously reported (discrepancies greater than 1 year were coded as failing to pass this question). Another question asked them to identify the capital of England (responses other than “London” failed to pass this question). Finally, participants were asked: “How often have you had a fatal heart attack?” Only one response option (“Never”) was correct,^5^ and selecting any other was coded as failing this question. Participants who failed one or more of these “catch” questions were excluded from further analyses (as explained above).

As always, decisions to not participate were coded as £0 donations. Two participants (both from the 5-mile run condition) reported exceptionally large donation amounts (£500 and £2000), so their data were excluded from the analyses.

### Results and discussion

The three meaningfulness ratings were highly correlated (all *r*s > .82) so we averaged them to obtain a single meaningfulness score for each participant. Furthermore, a pair of 2 (fundraiser type) × 2 (question order) ANOVAs, with donation amount and meaningfulness as the dependent variables, revealed only a main effect of fundraiser type (donations: *F*(1, 353) = 19.38, *p* < .001; meaningfulness: *F*(1, 353) = 5.55, *p* < .02). There were no main effects of question order (i.e., whether meaningfulness ratings or donation questions came first) or interactions between question order and fundraiser type (all *F*s < 1.60, ns). We therefore collapse across (i.e., ignore) question order in all subsequent analyses.

The reported likelihood of participating in the fundraiser was comparable across conditions: 58% wished to participate in the picnic and 51% in the 5-mile run (*χ*^2^(1) = 1.98, ns). However, participants in the painful–effortful fundraising condition offered to donate significantly more (*M* = £17.95) than participants in the easy–enjoyable fundraising condition (*M* = £5.74), *p*_perm_ < .001 (with 1000 iterations); *t*-test for unequal variances: *t*(224.61) = 4.56, *p* < .001, *d* = .61. Participants also judged the painful–effortful fundraiser as being significantly higher on meaningfulness (*M* = 6.46, *SD* = 2.15) than the easy–enjoyable one (*M* = 5.93, *SD* = 2.06), *t*(355) = 2.36, *p* < .02, *d* = .25. These effects of fundraiser type remained significant in a linear regression model with simultaneously entered controls for participant age, gender, willingness to participate in the event, having donated money to charity before, liking to run/jog for exercise, and having participated in a charity run/marathon before: *b* = £13.59, *t*(349) = 5.39, *p* < .001 and *b* = .64, *t*(349) = 3.34, *p* < .001 for donation amount and meaningfulness score, respectively.

To determine whether the effect of fundraiser type on donation amounts was mediated by perceived meaningfulness, we followed the approach recommended by Baron and Kenny (1986). First, we regressed donation amounts on fundraiser type and found that the painful–effortful fundraiser yielded larger donations (*β* = .23, *t*(355) = 4.38, *p* < .001). Second, we regressed judged meaningfulness on fundraiser type and found that the painful–effortful fundraiser was judged to be more meaningful (*β* = .12, *t*(355) = 2.36, *p* < .02). Third, we regressed donation amounts on judged meaningfulness, while controlling for fundraiser type, and found that judged meaningfulness predicted donations (*β* = .29, *t*(354) = 5.84, *p* < .001). This regression also revealed that the effect of fundraiser type on donations was smaller after controlling for meaningfulness (*β* = .19, *t*(354) = 3.82, *p* < .001). A Sobel test verified that this reduction in the effect was significant (*z* = 2.19, *p* < .03). Thus, perceptions of meaningfulness were found to partially mediate the relation between fundraiser type and donation amounts.

## EXPERIMENT 5

One possible boundary condition to the martyrdom effect concerns the nature of the charitable cause. A quick (and informal) sampling of charitable events reveals that painful–effortful fundraisers are mainly used to raise money for causes that involve human suffering, such as disease, poverty, and natural disasters. In contrast, causes that do not involve suffering (e.g., political candidates, museums, children's sports teams) are much less likely, it appears, to use sweat and tears as means to solicit contributions. This might be more than a coincidence: charities addressing human suffering may attract more donations when their fundraisers also involve suffering, in the form of painful–effortful events. Indeed, putting an ear to our intuitions tells us that running, walking, and biking great distances feel like legitimate ways to raise money for the victims of disasters, whereas a charity dance party with games, cake, and ice cream seems highly inappropriate in this case. Conversely, eating sweets seems like a more fitting way to help the Girl Scouts raise money than walking on shards of broken glass. The implication is that causes associated with human pain (e.g., the Red Cross) will generally benefit more from painful–effortful fundraisers than from easy–enjoyable ones but that this effect may dissipate, or even reverse, for causes associated with human enjoyment (e.g., the symphony orchestra). The nature of the cause may thus prove to be an important moderator of the martyrdom effect.

To examine this possibility, we used hypothetical scenarios similar to those in Experiment 1. Here, however, we independently varied the nature of the charitable cause and the fundraiser. For half the participants, the cause involved human suffering (helping feed starving children in the poorest countries). For the other half, the cause involved human enjoyment (building a new public park). Furthermore, the fundraising process was either painful–effortful (a 30-hour fast) or easy–enjoyable (an outdoor picnic).

To the extent that the martyrdom effect is moderated by the nature of the cause, as described above, we should observe a greater willingness to contribute with painful–effortful fundraisers (relative to easy–enjoyable ones) when the cause in question involves suffering but less so when it involves enjoyment. In other words, we predict a cause–fundraiser interaction.

### Method

#### Participants

We collected data from 184 US shoppers at a local mall (48% male; age range: 18–86, *M* = 34.75, *SD* = 14.73), who participated in exchange for compensation.

#### Procedure

These shoppers completed a pen-and-paper questionnaire, which presented them with hypothetical scenarios and choices in a 2 (cause = starving children vs. public park) × by 2 (fundraiser = fasting vs. picnic) between-subjects design, with participants assigned to one of the four conditions in alternating order.

As before, the survey asked participants whether they would participate in the fundraiser and, if so, how much they would donate to participate (with “no” responses coded as $0 donations). Data from 12 shoppers had to be excluded because two participants had difficulty reading English, nine were distracted and/or gave incoherent responses, and one had previously participated in a similar study. These excluded participants were all flagged by an experimenter who was blind to condition assignment. Removal of outliers using the 3 × IQR rule led us to exclude data from an additional 20 participants who reported exceptionally large donations. The final sample consisted of 152 participants.

### Results and discussion

The likelihood of participating differed significantly across the four conditions: *χ*^2^(3) = 21.35, *p* < .001, *ϕ =* 0.37. Participants were most likely to participate in the [public park + picnic] condition (88%), followed by the [starving children + picnic] condition (85%), then the [starving children + fasting] condition (69%), and least likely to participate in the [public park + fasting] condition (44%). The two picnic conditions did not differ in participation rates (*χ*^2^(1) < .08), and the difference between the two starving-children conditions was only marginally significant (*χ*^2^(1) = 2.74, *p* < .10). Every other pair-wise comparison between participation rates was significant (*p*s < .05). Overall, participation in the picnic conditions was higher than in the fasting conditions.

**Figure 3:**
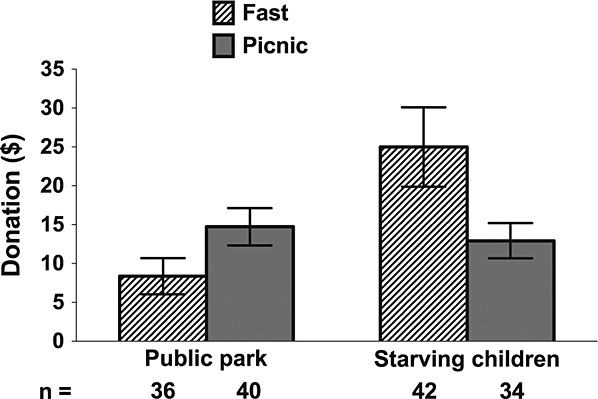
Mean donations as a function of cause and fundraiser type in Experiment 5. The means include nonparticipation responses, coded as $0 donations. Numbers underneath the bars indicate the sample size in each condition. Error bars represent ±1 standard error

However, mean donations (including $0 for nonparticipation) followed a very different pattern. Figure 3 presents the mean donations in each of the four conditions. A Kruskal–Wallis test revealed a significant difference between conditions, *χ*^2^(3) = 10.70, *p* < .02, *ϕ =* 0.27. To formally test the moderating role that the charitable cause might play in the martyrdom effect, we entered the cause and fundraiser variables into an ANOVA as independent factors. This 2 × 2 ANOVA revealed a main effect of cause, *F*(1, 148) = 4.61, *p* < .04, *η*^2^ = .03, such that (collapsing across fundraisers) participants in the starving-children conditions reported a greater willingness to donate (*M* = $19.59) than those in the public-park conditions (*M* = $11.71), *p*_perm_ < .02, unequal variances *t*-test: *t*(117.56) = 2.25, *p* < .03, *d* = .41. In addition, we found a significant cause–fundraiser interaction, *F*(1, 148) = 7.12, *p* < .009, *η*^2^ = .05. For the cause involving suffering (starving children), participants reported a greater willingness to donate when the fundraiser was painful–effortful (fasting: *M* = $24.99) than when it was easy–enjoyable (picnic: *M* = $12.93), *p*_perm_ < .03, unequal variances *t*-test: *t*(55.94) = 2.16, *p* < .04, *d* = .58. However, for the cause involving enjoyment (public park), the effect seemed to reverse: participants reported a greater willingness to donate when the fundraiser was easy–enjoyable (picnic: *M* = $14.73) than when it was painful–effortful (fasting: *M* = $8.36), *p*_perm_ < .04, *t*-test: *t*(74) = 1.89, *p* = .062, *d* = .44. In sum, we find that the martyrdom effect depends on the nature of the prosocial cause.

Prosocial causes involving human enjoyment may sometimes attenuate, rather than reverse, the martyrdom effect. In another study (not reported here) that crossed prosocial cause (curing muscular dystrophy vs. building a public park) with fundraiser type (5-mile run vs. picnic), we found that, although the martyrdom effect was reduced to non-significance when the cause shifted from suffering (muscular dystrophy) to enjoyment (public park), it did *not* reverse direction. Indeed, we still found a main effect of martyrdom across causes (an effect mainly driven by the muscular dystrophy conditions). Similarly, the absence of a cause involving human suffering may not always eliminate the martyrdom effect. In Experiment 2, the painful cold pressor condition yielded larger allocations to the collective than the painless control condition even though the cause in that case (fellow college students) neither involved human suffering nor seemed related to the fundraising “event” (the cold pressor task) in any other way.

These caveats notwithstanding, the results of Experiment 5 lend support to the hypothesis that the martyrdom effect can be moderated by the nature of the cause. Exactly why this occurs is not yet well understood. One possibility is that contributors experience more empathy for the recipients of charity when their (anticipated) fundraising experience brings them psychologically closer to these recipients (e.g., fasting for an extended time period may help one feel closer to the victims of famine). Increasing empathy in this way is likely to increase prosocial giving (Cryder & Loewenstein, 2010; Dickert, Sagara, & Slovic, 2010; Small, 2010). At the same time, this does not seem to explain the popularity of painful–effortful fundraisers that are largely detached (in terms of visceral experience) from their target causes, such as bike-a-thons to raise money for the hungry. Clearly, more research is needed to clarify the psychological processes involved.

## GENERAL DISCUSSION

In this paper, we have demonstrated a counterintuitive phenomenon: the addition of pain and effort can increase willingness to contribute to a prosocial cause. In economic parlance, we have shown that increasing the transaction costs of donating can boost transactions between donors and charities. The results of five experiments established the existence of a “martyrdom effect” (that the prospect of suffering to raise money for a charitable or collective cause leads to larger contributions), ruled out several confounds and alternative explanations, and identified a mediator and moderator of the effect.

Experiment 1 showed that people will donate more to a charity when they anticipate having to suffer to raise money (compared to when the fundraising process is expected to be easy and enjoyable). It also ruled out the possibility that people simply prefer painful–effortful fundraising events by showing that, in direct comparison, participants predominantly preferred attending the easier event.

Experiment 2 extended these findings to a laboratory context involving real money and actual pain. We found that participants in a public goods game made larger contributions to the collective when doing so was expected to be painful, yet this manipulation had no effect on their beliefs about what other players would do. This, combined with the fact that allocation decisions were unknown to other players, suggests that participants were not inferring the value of contributing from the presence of the painful task (Gneezy & Rustichini, 2000) nor trying to convey signals to each other or gain status within their group through their allocation choices, distinguishing the martyrdom effect from strategic altruism (e.g., Bénabou & Tirole, 2006; Hardy & Van Vugt, 2006). Experiment 2 further shows that the martyrdom effect impacts not just charitable giving but prosocial contribution decisions more generally.

Experiment 3 tested the hypothesis that the martyrdom effect reflects an attribute substitution strategy (Kahneman & Frederick, 2002), whereby people use the amount of pain–effort involved in fundraising as a cue to determine contribution worthiness. Contrary to this account, we found no relationship between the distance participants would have to run to raise money for charity and their willingness to contribute. This also distinguishes the martyrdom effect from theories that posit a monotonic or inverse U-shaped relationship between pain–effort and its impact (Bem, 1967; Festinger, 1957; Locke & Latham, 2006; Trope & Fishbach, 2000).

Experiment 4 showed that feelings of meaningfulness partially mediate the relationship between fundraiser type and contribution amounts. This suggests, in line with our hypothesis, that painful–effortful fundraisers make the experience and act of contributing seem more meaningful for people, thereby increasing their willingness to contribute. The fact that we found partial (as opposed to full) mediation further suggests that other factors may also influence contribution amounts.

Finally, Experiment 5 demonstrated that the nature of the prosocial cause can moderate the martyrdom effect: the effect was found when the cause was associated with human suffering but not when it was associated with enjoyment. This result is reminiscent of stimulus–response compatibility effects, prevalent in perception, cognition, and decision making (e.g., Proctor & Reeve, 1990; Shafir, 1995; Slovic, Griffin, & Tversky, 1990), where compatibility between stimulus and response or between an attribute and a response scale can increase the weight or attention assigned to the stimulus or the compatible attribute. In the case of Experiment 5, a fundraising method compatible with the cause (i.e., where both might involve physical suffering) apparently increased willingness to contribute to the cause. However, what we report is not a standard compatibility effect because (i) the response mode was not varied in our experiments and (ii) we found that the martyrdom effect was more attenuated than reversed when the prosocial cause was associated with human enjoyment (as opposed to human suffering). Nevertheless, an account based on compatibility (broadly defined) seems worthy of further investigation.

Critically, in every one of these experiments, participants reported the amount they wanted to contribute *before* they experienced any pain–effort associated with the contribution process. This distinguishes the martyrdom effect from cognitive dissonance theory (Festinger, 1957) and self-perception theory (Bem, 1967), which predict that overcoming an obstacle in order to achieve a goal can lead people to value that goal more than they did before completing the challenge. Although these theories could explain a greater willingness to donate to charity *after* completing a painful–effortful fundraising event, they do not explain the greater willingness to donate *before* participating in the challenging endeavor. Indeed, it is this *prospect* of pain–effort increasing prosocial behavior that constitutes the core finding of the present paper. Thus, the martyrdom effect differs from these theories in at least two major ways: (1) the *amount* of pain–effort involved does *not* matter, only its presence vs. absence and (2) the effect is prospective. An intriguing possibility is that prosocial contributions might be sensitive to retrospective evaluations of pain–effort quantity (as the above theories would predict) but less so to prospective evaluations (as we have found).

Table 1 summarizes the results of all experiments (reported in this paper) that compared contributions associated with painful–effortful and easy–enjoyable fundraisers. As this table shows, we found strong positive effects of adding pain and effort, with effect sizes ranging from medium to large (averaging .62) and large relative increases in contributions (averaging 94%).

**Table 1:** Summary of results obtained across experiments when comparing contributions offered in painful–effortful and easy–enjoyable fundraisers

		Effect size	Sample size	
				
	Relative increase in mean contributions (%)	*d*	*N*	Charity/cause
Experiment 1a (endurance run vs. picnic)	72	0.70	96	Asian tsunami
Experiment 1b (endurance run vs. picnic)	59	0.51	96	Hurricane Katrina
Experiment 2 (cold pressor vs. control)	31	0.70	36	Public pool (collective)
Experiment 4 (endurance run vs. picnic)	213	0.61	357	Victims of war and genocide
Experiment 5 (30-h fast vs. picnic)	93	0.58	76	Starving children in poor countries
Average [total]	94	0.62	[661]	

Note. In all these comparisons, the mean amount contributed was greater in the painful–effortful condition than in the easy–enjoyable one.

In all but one of these studies, the likelihood of contributing did not significantly differ across conditions. The martyrdom effect therefore seems to primarily influence *how much* people contribute rather than *whether* they decide to contribute in the first place. A full account of this phenomenon will have to explain not so much why people agree to participate in painful–effortful fundraisers but rather why their contributions increase in those contexts. Still, we did observe a persistent, if mild, tendency for participants to be less likely to participate in painful–effortful fundraisers than in easy–enjoyable ones. Although it failed to reach significance in all but one experiment, this trend suggests that painful–effortful fundraisers are likely to deter a small fraction of potential contributors (as we might expect). At the same time, we find that these events more than make up for this loss by stimulating larger contributions from those who do participate (not to mention their sponsors). Because decisions about how much to contribute appear to be somewhat insensitive to the amount of pain–effort involved (see Experiment 3), charities would do well to organize fundraisers that strike the right balance between being painful–effortful enough to be perceived as challenging (thus attracting larger contributions), and not so difficult as to deter large numbers of potential donors.

Because of ethical considerations (the use of pain and effort) and budget constraints (the costs of organizing charity fundraisers), many of our studies involved hypothetical decisions. A potential concern, therefore, is that reported donations were unrealistically high. This concern was addressed in Experiment 2, which involved real contribution decisions (with real pain and money at stake). Even putting aside Experiment 2, however, this paper is not so much concerned with the absolute level of contributions as it is with the *difference* between contributions associated with painful–effortful and easy–enjoyable fundraisers. And throughout our studies, participants reported a greater willingness to contribute (to causes involving human suffering) when their contributions were associated with pain–effort. It is unclear how the hypothetical nature of their choices could have produced this observed difference. Even if contemplating hypothetical donations tends to upwardly bias reported willingness to contribute by some amount (or proportion), there is the added and persistent fact that reported amounts were larger for painful–effortful fundraisers. Therefore, one would still need to explain why any potential bias associated with hypothetical contribution decisions would be greater for painful–effortful contribution processes.

Several alternative accounts of the martyrdom effect might be worth considering. As explained above, our results are incompatible with a number of existing theories, both in economics (i.e., rational choice theory) and psychology (e.g., cognitive dissonance theory and self-perception theory). However, there are (as always) still other explanations that we have not discussed. For example, people might contribute more when doing so is expected to be painful–effortful as a way to justify their (future) suffering. Perhaps donating $1 in order to run 5 miles and raise one additional dollar appears to trivialize the anticipated pain and effort, whereas large contributions make the physical sacrifice seem worthwhile. But this line of reasoning (first agreeing to participate in a painful–effortful task then adjusting one's contributions to justify this puzzling behavior) is rather strange! Furthermore, this account would predict that increasing the amount of anticipated pain–effort should further increase prosocial contributions (i.e., the more someone expects to suffer, the more he/she would need to contribute to “rationalize” this pain–effort). Yet, as Experiment 3 shows, we find no relationship between the amount of anticipated pain–effort and contributions. Another possibility might be a “cultural” one: because painful–effortful charity fundraisers, such as marathons and bike-a-thons, typically attract large contributions (relative to many pain-free alternatives, such as door-to-door fundraising), donors may believe they are expected to contribute more when doing so is painful–effortful. Of course, this observation only begs the question of why it is that painful events typically attract more contributions than painless ones; and as Experiment 2 showed, the martyrdom effect occurs even outside the context of charity fundraising, under no such cultural expectations.

## IMPLICATIONS AND FUTURE DIRECTIONS

The martyrdom effect may have important implications for the design of charity fundraisers. Standard normative theories, which assume that people are motivated to avoid pain and effort, would prescribe making the fundraising experience as easy and enjoyable as possible in order to attract more participants and increase donation giving. In contrast, our results suggest that fundraising is best achieved by making the task of raising money challenging for participants. There are, of course, limits to what donors are willing to suffer for a cause, but the main point is that challenging donors is likely to contribute to the success of a charity fundraiser, whereas catering to basic hedonic desires may backfire, especially when the cause in question involves human suffering.

The martyrdom effect may also help to explain some puzzling phenomena in the psychology literature. Liu and Aaker (2008; see also Liu, 2010), for example, recently found that willingness to donate to a charitable cause increased if people first thought about donating time to the cause. Donating time for charity often involves some amount of effort, so if thinking about donating time leads people to envision themselves exerting effort for a cause, then the “time-ask effect” motivating greater giving may be a special case of the martyrdom effect. Similarly, Liu and Aaker's finding that donations decreased when potential donors first thought about donating money may be attributable to perceptions of lesser effort when people envision making only monetary contributions. Further research on what comes to mind when people think about contributing time versus money may help clarify these connections.

Many interesting questions about the martyrdom effect remain to be answered; for example, whether it extends to other choice domains or whether the amount of pain–effort that people are willing to endure for a cause depends on the nature of the cause or on donor dedication. We might also ask whether the martyrdom effect extends beyond the self to another; that is, are we also more motivated to contribute when it is someone else who is going to suffer for a valued cause? In a separate set of studies (Olivola & Shafir, 2011), we have found that people are indeed more willing to sponsor a friend's fundraising efforts if he/she is going to exert effort and experience pain in the process (compared with when his/her fundraising experience is less painful–effortful).

## CONCLUSION

Our goal in this paper was to explore the effects of (prospective) pain and effort on contributions to prosocial causes generally, with charitable fundraising providing a useful context in which to examine these broader questions. However, it should be noted that our examination of the martyrdom effect extended beyond the specific context of charitable giving to include other prosocial contribution decisions, such as the public goods game in Experiment 2. Research into the psychology of martyrdom has the potential to provide new theoretical insights into decision making and behavior, as well as transform the way we view motivation. Currently, the dominant view is that pain and effort are strong deterrents. As this paper demonstrates, however, human motivation is more complex. People often willingly challenge themselves by enduring pain and effort while working toward a desired goal, even when they do not have to. This goal, furthermore, can extend beyond one's own self-interest to the well-being of others. Indeed, we find not only that people are willing to participate in painful–effortful events and give away their money to aid anonymous others but that the prospect of experiencing pain and exerting effort for a prosocial cause can actually lead to greater altruism! The fact that people will make sacrifices (both physical and financial) for a cause, even when they stand to gain nothing tangible in return, leads us to conclude that the motivation to suffer for a cause deserves further study.

## Footnotes

1 We thank Talya Miron-Shatz for suggesting this scenario.

2 Reintroducing these extreme outliers into the analysis actually *increases* the size of the observed difference in mean reported contributions between the two conditions. However, doing so also increases the standard deviations, so the comparison is no longer significant.

3 For simplicity of word usage, we will often refer, in our results and discussion sections, to “the average amount contributed,” “mean contributions,” and the like, even though participants were not always asked to make real contributions. The potential limitation of asking for hypothetical contributions (or actual reported intentions to contribute) is addressed in Experiment 2, where participants made real contribution decisions (with real pain and money at stake).

4 This task was even more painful than the one used by Kahneman, Fredrickson, Schreiber, & Redelmeier (1993) in their classic study of retrospective evaluations of painful episodes. Their participants immersed just one hand in either 14°C water for 60 seconds or in 14°C water for 60 seconds followed by another 30 seconds in water that was gradually heated from 14°C to 15°C.

5 The “fatal heart attack” catch question was adapted from a similar one used by Paolacci, Chandler, and Ipeirotis (2010).

## APPENDIX: SEPARATE-EVALUATION SCENARIOS PRESENTED IN EXPERIMENT 1A

*Note*. The wording that differed across manipulations is presented in brackets. Wording unique to the ‘charity picnic’ condition is italicized, while wording unique to the ‘charity run’ condition is underlined.

## Authors' biographies

**Christopher Y. Olivola** is currently a Newton International Fellow in the Behavioural Science Group at the Warwick Business School (University of Warwick). His research interests span several related areas, including decision making, behavioral economics, social cognition, and experimental philosophy. He received his BA from the University of Chicago and his MA and PhD from Princeton University.

**Eldar Shafir** is the William Stewart Tod Professor of Psychology and PublicAffairs in the Department of Psychology and the Woodrow Wilson School of Public and International Affairs at Princeton University. His research focuses on decision making, with a particular interest in behavioral economics and policy. He received his PhD from MIT in 1988 and was a postdoctoral scholar at Stanford University.

## Authors' addresses

**Christopher Y. Olivola**, University of Warwick, UK.

**Eldar Shafir**, Princeton University, USA.
